# Superannuation under the Poor Law

**Published:** 1899-04-22

**Authors:** 


SUPERANNUATION UNDER THE POOR LAW.

"United Service" writes: The thanks of all interested in Poor Law work are due to you for the excellent *résumé* of the present state of the superannuation question as given in a recent number of The Hospital. No doubt the Poor Law Officers' Superannuation Act, 1896, was a step in progress, but the consequences of that Act on promotion are only now beginning to be felt. Boards are hesitating to appoint officers who have ten or more years of service to their record, as, if such officers break down, they draw their pensions entirely from the funds of the last union they serve. Instead of hundreds of little funds scattered all over the country, each jealously guarded by a Board, a central superannuation fund would meet all requirements, obviate all difficulties about promotion, and should there be any deficit it could easily be met by a contribution from each union on the basis of rateable value. The Poor Law service is rapidly increasing in importance, and it seems incredible that the broad principles on which the pension funds of the Civil Service, police, railway companies, &c., are based should not be extended to the service which cares for the poor of the State.

